# Antiproliferative Activity and Apoptosis Inducing Mechanism of *Anthocephalus cadamba* on Dalton’s Lymphoma Ascites Cells

**Published:** 2016

**Authors:** Narayan Dolai, Aminul Islam, Pallab Kanti Haldar

**Affiliations:** a *Department of Pharmaceutical Technology, Jadavpur University, Kolkata, India. *; b *Research and Development Centre, Natreon Inc, Salt Lake City, Kolkata, India.*

**Keywords:** *Anthocephalus cadamba*, DLA, Antitumor, Apoptosis

## Abstract

The purpose of this investigation was to evaluate the antiproliferative and apoptogenic mechanistic studies of methanol extract of *Anthocephalus cadamba *(MEAC) on Dalton’s lymphoma ascites (DLA) cells treated mice. Determination of antiproliferative activity was performed by using different DLA cells (2×10^6^ cells, i.p.) inoculated mice groups (*n *= 12). Groups were treated for 14 consecutive days with MEAC at the doses of 200 and 400 mg/Kg b.w. respectively. The mechanism of antiproliferation activity of MEAC was investigated through morphological studies by acridine orange (AO)/ethidium bromide (EB) double staining method. Comet assay was estimated to check the DNA damage induced apoptosis property. Furthermore, flow cytometry (FACS) was used to quantitatively detect the apoptotic rate by double labeling techniques using Annexin-V FITC/propidium iodide staining analysis and apoptotic proteins expression done by western blotting assay method. MEAC exhibited significant (*p*<0.01) decrease the tumor volume, viable cell count, tumor weight and elevated the life span of DLA tumor bearing mice. Analysis of AO/EB staining and flow cytometry showed that MEAC possessed apoptosis induced antitumor activity on DLA cells in a dose dependant manner. Dose dependent induction of DNA damage on DLA cells were observed after MEAC treatment, which was evident from the appearance of comet tail length. Pro-apoptotic gene, Bax was up-regulated and down-regulation of the Bcl-2/Bax ratio, suggesting that Bcl-2 family involved in the control of apoptosis. Experimental results revealed that MEAC possess potent antitumor activity *via* induction of cancer cell apoptosis mechanism.

## Introduction

Cancer is perhaps the most progressive and devastating disease posing a threat of mortality to the entire world despite significant advances in medical technology for its diagnosis and treatment. Recently, considerable attention has been focused on identifying natural occurring chemopreventive substance capable of inhibiting, retarding, or reversing the process of multistage carcinogenesis (1). 

It is now well recognized that apoptosis is a mode of cell death used by multi-cellular organisms to eradicate cells in diverse physiological and pathological settings. Recent evidence also shows that suppression of apoptosis by tumor promoting agents in pre-neoplastic cells is an important mechanism in tumor promotion (2). In this context, it is noteworthy that apoptosis-inducing ability seems to have become a primary factor in considering the efficacy of chemo-preventive agents.

*Anthocephalus cadamba *(Roxb.) Miq. (Rubiaceae) is commonly known as “Kadamba”, which is frequently found in moist deciduous evergreen forests and widely distributed throughout the greater part of India (3). Various plant parts are used as folk medicine in the treatment of tumor, inflammation, fever, hematological diseases, skin diseases, leprosy and hypoglycemic agent (3, 4). Until now, scientific bioactivity determination studies have revealed its antimalarial (5), antihepatotoxic (6), anti-inflammatory (7), antidiabetic (8), antioxidant, wound healing and antimicrobial activities (3). The antitumor activity and *in-vivo* antioxidant status of methanol extract *A. cadamba* on Ehrlich ascites carcinoma treated mice was reported in our previous experiment (9). Here we report the dose-dependent apoptogenic effect of MEAC on Dalton’s lymphoma ascites (DLA) cells treated mice. In the present study, we addressed the relevance of morphological detection, DNA damage by comet assay, flow cytometric analysis and Bax and Bcl-2 using western blotting of MEAC induced DLA cells apoptosis.

## Experimental

*Plant material and extraction*

The stem bark of *A. cadamba *(Roxb.) Miq. (Rubiaceae) was collected from the middle hill region of Sikkim in the month of September 2010 and authenticated by Botanical Survey of India, Gangtok, India. A voucher specimen (SHRC-5/5/2010/Tech.47A) was deposited at Phytotherapy and Pharmacology Research Laboratory, Department of Pharmaceutical Technology, Jadavpur University, Kolkata. First the collected stem bark was shade dried at room temperature for 7 days and then powdered in a mechanical grinder. Next, the powdered plant material (1 Kg) was successively extracted by methanol using Soxhlet extraction apparatus. Then solvent was completely evaporated under reduced pressure, *in vacuo* at 40 °C to render the methanol extract (MEAC; 200 g; 20.0% w/w). 

*Phytochemical analysis of MEAC*

*Qualitative screening*

The presence of various phytoconstituents such as glycosides, steroids, saponins, alkaloids, tannins, phenolics and flavonoid were analyzed by using standard protocols (10).

*Quantification of total phenolic and flavonoid contents*

The quantities of phenolics and flavonoids compound were measured by the method described in our previous study (11). The Folin–Ciocalteu method and aluminium trichloride colormetric assay were applied for the determination of phenolics and flavonoids compound, respectively.

*Animals*

Swiss albino mice of about 8 weeks of age with an average body weight of 20-25 g were used for the experiment. The mice were grouped and housed in poly acrylic cages (38 cm × 23 cm × 10 cm) with not more than six animals per cage.

 The animals were maintained under standard laboratory conditions (temperature 25-30 °C and 55-60% relative humidity with dark/light cycle 14/10 h) and were allowed to free access of standard dry pellet diet and water *ad libitum*. The mice were acclimatized to laboratory conditions for 7 days before commencement of the experiment. 

*Acute toxicity study*

The acute oral toxicity of MEAC in Swiss albino mice was performed as per OECD guideline 425 (OECD, 2008). The extract was safe up to the dose of 2 g/Kg b.w. p.o. for mice. Generally 1/5th to 1/10th of the lethal dose has taken for effective dose calculation. So, 200 and 400 mg/Kg b.w. doses were used in the present study (9). 

*Cell culture*

DLA cells were obtained from Chittaranjan National Cancer Institute, Kolkata, India. Ascitic fluid was drawn out from DLA tumor bearing mouse at the log phase (days 7–8 of tumor bearing) of the tumor cells. The DLA cells were maintained *in-vivo* in Swiss albino mice by intraperitoneal transplantation of 2×10^6^ cells per mouse after every 10 days and it is used for both *in-vivo *and *in-vitro* study. 

*Cell viability assay*

Cell viability was determined by using the3-(4,5-dimethylthiazol-2-yl)-2,5-diphenyltetrazolium (MTT) assay (12). Briefly, 0.1 mL of cell suspension was seeded in 96-well plates (Greiner, Frickenhausen, Germany) with a seeding density of 1×10^4^ cells/well. The cells were treated with different concentrations of MEAC (10, 25, 50, 100, 150 and 200 μg/mL) and incubated for 24 h at 37 °C, 5% CO_2_ with 98% relative humidity. After incubation, 20 μL of MTT (5 mg/mL) in phosphate buffered saline (PBS) were added to each well and the plates were incubated for 4 h at 37 °C. The colored formazan crystals which were produced from MTT were dissolved in 150 μL of dimethyl sulfoxide (DMSO) and the absorbance was measured at a wavelength of 570 nm by ELISA plate reader. All determinations were carried out in triplicate. Concentrations of MEAC showing 50% reduction in cell viability (ie, IC_50_ values) were then calculated.

*Treatment schedule* *for assessment of antitumor potential*

Swiss albino mice (20–25 g) were divided in to four groups (*n *= 12). Except Group-I all the animals in each groups were being injected DLA cells (2×10^6^ cells/mouse, i.p.). This was marked as day ‘0’. Group-I served as normal saline control (5 mL/Kg, i.p.) and group-II served as DLA control. After 24 h, DLA transplanted Group-III and IV were being injected MEAC (200 and 400 mg/Kg b.w, i.p.) once daily for 14 consecutive days (9). After administration of last dose, 6 mice from each group were kept fasting for 18 h and blood was collected by direct cardiac puncture for the estimation of hematological parameters determination and then those animals were sacrificed for collection of DLA cells to check the apoptogenic properties of stem bark of *A. cadamba*. Rest of the animals in each groups were kept alive with food and water *ad libitum* to check percentage increase in life span of the tumor host to determine the mean survival time (MST).

Antitumor activity of MEAC was assessed by observation of changes with respect to the following parameters (13).

*Tumor volume and weight*

The mice were dissected and the ascitic fluid was collected from the peritoneal cavity. Volume of the fluid was measured by taking it in a graduated centrifuge tube and expressed in milliliter (mL). Tumor weight was measured by taking the weight of the mice before and after the collection of the ascitic fluid from peritoneal cavity and expressed in gram (g).

*Percentage increase life span (% ILS)*

The effect of MEAC on tumor growth was monitored by recording the mortality of the experimental mice. Percentage increase in life span (%ILS) was calculated by the following formula. 

Mean survival time (MST) in days = (Day of the first death + Day of the last death) / 2

ILS (%) = [(MST of the treated group / MST of the control group) – 1] × 100

*Tumor cell (Viable/nonviable) count*

The ascitic fluid was taken in a WBC pipette and diluted upto 20 times with PBS solution. Then a drop of the diluted cell suspension was placed on the Neubauer’s counting chamber and the numbers of cells in the 64 small squares were counted. 

The viability and non-viability of the cell were determined by trypan blue assay. The cells were stained with trypan blue (0.4% in normal saline) dye. The cells that did not take up the dye were viable and those that took the dye were nonviable. These viable and nonviable cells were counted by using the under-scribbled formula.

Cell count = (Number of cells × dilution factor) / (Area × thickness of liquid film)

*Hematological parameters*

Collected blood was used for the estimation of hemoglobin (Hb), red blood cell (RBC) and white blood cell (WBC) count by standard procedures (14).

*Detection of apoptosis*

Isolation of DLA from mice peritoneal cavity 

The DLA cells were isolated from the peritoneal cavity of tumor-bearing mice (control or treated). 3 mL of sterile PBS was injected into the peritoneal cavity of the mice and the peritoneal fluid containing the tumor cells was withdrawn, collected in sterile Petri dish and incubated at 37 °C for 2 h. The cells of macrophage lineage adhered to the bottom of the Petri dishes. The non-adherent population was aspirated out gently and repeatedly washed with PBS. The viability of DLA was assessed by trypan blue exclusion test. The viable DLA cells were processed for further experiments (15).

*Acridine orange (AO) and ethidium bromide (EB) double staining*

DNA binding dyes AO and EB (Sigma Aldrich, USA) were used for the morphological detection of apoptotic and necrotic cells (16). DLA cells (1×10^6^) were collected from sacrificed mice. The cells were detached, washed by cold PBS and then stained with a mixture of AO (100 μg/mL) and EB (100 μg/mL) at room temperature for 5 min. The stained cells were observed by a fluorescence microscope (Leica DM 3000, Germany) at 40X magnifications. The cells were divided into four categories as follows: living cells (normal green nucleus), early apoptotic (bright green nucleus with condensed or fragmented chromatin), late apoptotic (orange-stained nuclei with chromatin condensation or fragmentation) and necrotic cells (uniformly orange-stained cell nuclei). In each experiment, more than 300 cells/sample were counted for % of apoptotic cells count.

*Comet Assay*

The extent of DNA was quantified by alkaline single cell gel electrophoresis (SCGE), also known as the comet assay. Briefly, cells suspended in 0.5% (w/v) low melting agarose were layered over a frosted microscopic slide coated with a layer of 1% normal melting agarose. The slides were left in a lysing solution overnight at 4 °C. Electrophoresis was carried out for 30 min (280 mA, 20 V) at 4 °C. The slides were washed thrice with neutralizing buffer (Tris 0.4 M, pH 7.5), stained with EB, examined under a fluorescence microscope (Leica DM3000, Germany) and subjected to image analysis using CometScore software (17).

*Flow cytometric analysis (FACS) assay*

Assay was performed using procedure described in the reagent-kit purchased from BD Biosciences and protocol was followed by manufacturer’s instruction. To distinguish between apoptosis and necrosis, in a double labeling system, DLA cells from untreated or MEAC treated tumor-bearing mice were washed twice with cold PBS and then re-suspended in 1X binding buffer at a concentration of 1×10^6 ^cells/mL. Then 100 µL of the cell suspension was transferred to the 5 mL culture tube. Afterward, added 25 µL of Annexin V-FITC and/or propidium iodide (PI) solution to the cell suspension. The cell were gently mixed by vortexing and incubated for 15 min at 37 °C. Then 400 µL of 1X binding buffer was added to each tube and analyzed by FACS within one hour using flowcytometer (BD LSRFortessa^TM^ Cell analyzer, USA).

*Analysis of protein expression by Western blotting*

DLA lysate was loaded into a 10% Sodium dodecyl sulfate (SDS)-polyacrylamide gel. After electrophoresis, the gel was transferred to nitrocellulose membrane and blocked with 5% Bovin serum albumin (BSA) in 1X tris buffer saline (TBS). The membrane was then incubated with specific primary antibody of anti-Bax, anti-Bcl-2 and β-actin (1:1000) for overnight at 4 °C. The protein of interest was visualized by treating with alkaline phosphatase (ALP) conjugated specific secondary antibody. The target protein band was then visualized using bromochloroindolyl phosphate (BCIP) and nitrobluetetrazolium (NBT) substrates. Equal loading of protein in each lane was established by β-actin antibody probing (1). 

*Statistical analysis*

Data was statistically analyzed by using one way analysis of variance (ANOVA) followed by Dunnett’s post hoc test by GraphPad Prism software (Version 5.0, GraphPad Prism software Inc., San Diego, CA).* p*<0.01 was considered as statistically significant. 

**Table 1:** Phytochemical analysis of MEAC

**Fractions**	**Steroid**	**Glycoside**	**Alkaloid**	**Saponin**	**Tannin**	**Phenolic** **mg GAE/g**	**Flavonoid** **mg QE/g**
MEAC	+	+	+	+	-	164.75 ± 3.8	51.20 ± 2.1

+: present; -: absent; GAE: gallic acid equivalent; QE: quercetin equivalent; Values are represents the mean of triplicate experiments (mean ± S.E.M).

**Table 2:** Effect of MEAC on tumor volume (ml), tumor weight (g), viable (Cells×10^7 ^cell/mL) and nonviable cell count (Cells×10^7 ^cell/mL), median survival time (MST), percentage increase life-span (%ILS) and hematological parameters like RBC (cells×10^6^/µL), WBC (cells×10^3^/µL) and Hb content (g/dL) in DLA cells bearing mice

Parameters	Normal control (5ml/kg)	DLA control (2× 10^6 ^cell/ml)	DLA + MEAC (200 mg/kg)	DLA + MEAC (400 mg/kg)
**Tumor volume**	-	2.99 ± 0.40	1.66 ± 0.28^ b,^^*^	1.26 ± 0.15^ b,^^*^
**Tumor weight**	-	2.85 ± 0.21	1.37 ± 0.18^ b,^^*^	0.91 ± 0.07^ b,^^*^
**Viable cell**	-	9.12 ± 0.45	3.99 ± 0.39^ b,^^*^	1.76 ± 0.28^ b,^^*^
**Nonviable cell**	-	0.38 ± 0.07	1.24 ± 0.15^ b,^^*^	3.03 ± 0.72^ b,^^*^
**MST (days)**	-	20.00	30.50	37.00
**% ILS**	-	00	52.50	80.00
**RBC**	5.63 ± 0.19	2.46 ± 0.60 ^a,*^	4.01 ± 0.18 ^b,^^*^	4.96 ± 0.42^ b,^^*^
**WBC**	5.11 ± 0.39	10.16 ± 1.05 ^a,*^	7.43 ± 0.31^ b,^^*^	6.13 ± 0.86^ b,^^*^
**Hemoglobin**	11.50 ± 1.01	5.12 ± 0.91 ^a,*^	9.05 ± 0.90^ b,^^*^	9.91 ± 0.80^ b,^^*^

Values are represented as mean ± S.E.M, of three independent experiments (*n*=3).

a DLA control group *vs *normal group,

b treated groups *vs *DLA control group,

* *p*<0.01.

**Figure 1:**
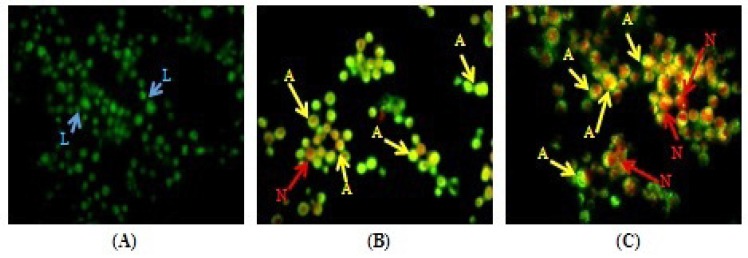
Morphological changes in DLA cells after 14 days treatment with MEAC extract. For DLA control group (A), MEAC 200 mg/kg (B) and MEAC 400 mg/kg (C). The cells were stained with acridine orange and ethidium bromide. Blue arrows next to "L" point to live cells; Yellow arrows next to "A" indicate apoptotic cells; and Red arrows next to "N" indicate necrotic cells (magnification at 40X).

**Figure 2:**
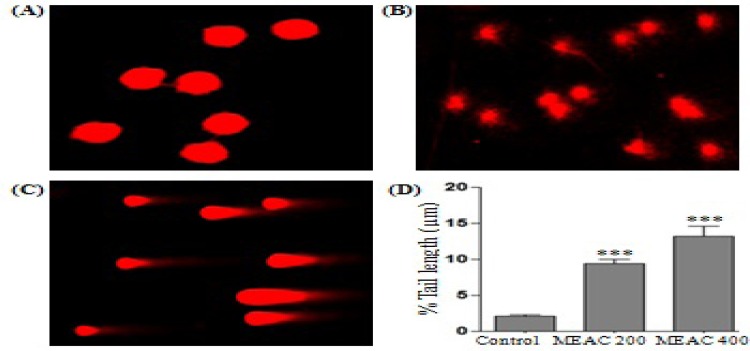
The DNA damage was measured by comet assay after treatment of DLA cells with MEAC extract. The cells were left untreated (A) or treated with MEAC 200 mg/kg (B) or MEAC 400 mg/kg (C). The extent of DNA damage was expressed in terms of comet % tail length (D). Data are the mean ± SD from three replicate measurements. Treated groups *vs.* DLA control group, ***p<0.001

**Figure 3:**
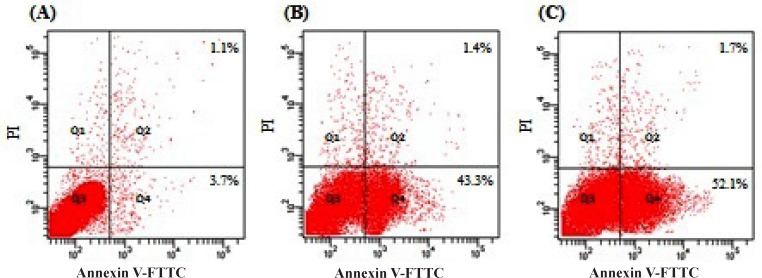
Flow cytometry analysis for apoptosis inducing activity of MEAC on DLA cells were labeled with PI and Annexin-V FITC Fluos and then fixed and analyzed on a Flowcytometer. DLA control group (A), MEAC 200 mg/kg (B) and MEAC 400 mg/kg (C). Dual parameter dot plot of FITC-fluorescence (x-axis) versus PI-fluorescence (y-axis) has been shown in logarithmic fluorescence intensity. Quadrants: lower left, live cells; lower right, apoptotic cells; upper right, necrotic cells

**Figure 4:**
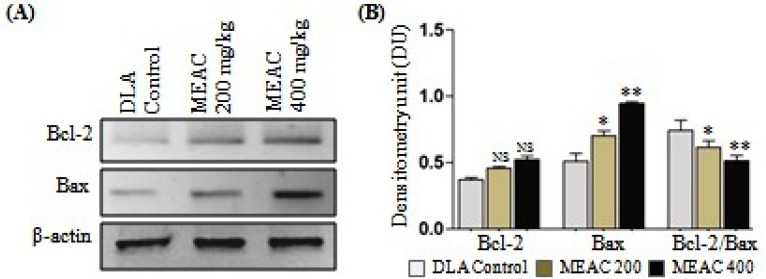
DLA lysates [Lane 1- DLA control; Lane 2- MEAC treated 200 mg/kg and Lane 3- MEAC treated 400 mg/kg] were subjected to western blot analysis. Pro-apoptotic proteins Bax and anti-apoptotic protein Bcl-2 and visualized by ALP-conjugated secondary antibody. The β-actin band confirmed equal protein loading (A). Quantitative bar diagram showing Bcl-2/Bax expression and ratio (B), Data are the mean ± SD from three replicate measurements. Treated groups *vs.* DLA control group, NS= non significant, *p<0.05 and **p<0.01

## Results

Qualitative screening of MEAC revealed the presence of steroids, glycosides, saponins, alkaloids, phenolics and flavonoids. Total phenolic contents were found 164.75 ± 3.8 mg gallic acid equivalent/g, and flavonoid compounds were found to be 51.20 ± 2.1 mg of quercetin equivalents/g of MEAC (Table 1).

The MEAC were able to reduce viability of the DLA cells in a dose-dependent manner, and the IC_50_ value was found to be 90.45 ± 3.94 µg/mL.

Antitumor activity of MEAC against DLA tumor bearing mice was assessed by the parameters such as tumor volume, tumor weight, cell count (viable and non-viable), mean survival time and % increase in life span. The tumor volume, tumor weight and viable cell count were found to be significantly (*p*<0.01) increased and non-viable cell count was significantly (*p*<0.01) declined in DLA control animals, when compared with normal control animals (Table 2). Administration of MEAC at the doses of 200 and 400 mg/Kg significantly (*p*<0.01) decreased the tumor volume and viable cell count. Non-viable cell count was significantly (*p*<0.01) higher in MEAC treated animals when examined with respect to DLA control animals. Furthermore, the median survival time was increased to 30.5 days (% ILS = 52.50) and 37.00 days (% ILS = 80.00) on administration of MEAC at the doses of 200 and 400 mg/Kg respectively.

There was significantly (*p*<0.01) elevated level of WBC and significantly (*p*<0.01) reduced level of RBC and hemoglobin (Hb) in DLA control group as compared to normal control group (Table 2). But, treatment with MEAC at the doses of 200 and 400 mg/Kg in DLA bearing mice significantly (*p*<0.01) increased both the RBC count and Hb content while WBC count was deduced significantly (*p*<0.01) when compared with the DLA control group. 

Morphological features of apoptosis such as chromatin condensation, nuclear fragmentation, alterations in the size and shape of cells, as revealed by fluorescence microscopic analysis, were observed predominantly after MEAC treatment. AO penetrates into living cells, emitting green fluorescence after intercalation into DNA. The second dye, EB emits red fluorescence in the cells with an altered cell membrane. Treatment with MEAC at doses 200 and 400 mg/Kg has shown the number of apoptotic cells 41.66 ± 1.58 and 45.33 ± 2.82% (Figure 1B and C).

Treatment dependent DNA damage in DLA cells was observed after MEAC treatment compared with the untreated DLA control (Figure 2A), MEAC-induced DNA damage even at a low concentration (200 mg/Kg), as indicated by the presence of the DNA tail (Figure 2B). A greater % tail length (distance from DNA head to the end of DNA tail), more extensive were the DNA damage (Figure 2C). Experimental results revealed that MEAC induced substantial DNA damage which was evident from the appearance of comet length (9.42 ± 1.67 and 13.17 ± 3.71 µM) in MEAC (200 and 400 mg/Kg) treated cells (Figure 2D) with respect to the untreated control (2.11 ± 0.48 µM). This result could connote that MEAC induced apoptosis followed by DNA damage. 

Cells stained with Annexin V-FITC and PI were classified as necrotic cells (the upper left quadrant; Annexin^−^/PI^+^), late apoptotic cells (the upper right quadrant; Annexin^+^/PI^+^), intact cells (the lower left quadrant; Annexin^−^/PI^−^) or early apoptotic cells (the lower right quadrant; Annexin^+^/PI^−^). The result showed that treatment at different doses of MEAC could quantitatively induce apoptosis in DLA cells (Figure 3A, B and C). Apoptosis ratios for MEAC at the doses 200 and 400 mg/Kg were found to be 43.3% and 52.1%, respectively.

It is well recognized that various pro- and anti-apoptotic proteins are hugely responsible for induction of apoptosis. The expression of pro-apoptotic proteins Bax and as well as anti-apoptotic protein Bcl-2 in our mice model were examined to study the effect of the MEAC at the doses 200 and 400 mg/Kg on DLA cells. We observed, in DLA the levels of Bax protein expression increased significantly after MEAC treatment of the tumor host, whereas, no significant change in the level of Bcl-2 was observed (Figure 4A). Interestingly, the Bcl-2/Bax ratio was significantly decrease follwed by MEAC treatment on DLA cells (Figure 4B). 

## Discussion

The present study was carried out to evaluate the antiproliferative activity and apoptosis inducing mechanism of *Anthocephalus cadamba* on Dalton’s lymphoma ascites cells treated mice. Dalton’s ascites lymphoma is transplantable, poorly differentiated malignant tumor which appeared originally as lymphocytes in a mouse. It grows in both solid and ascitic forms. From the present experiment, it is limpid that treatment with MEAC at the doses 200 and 400 mg/Kg significantly reduced the tumor volume, tumor weight, tumor cell count (viable and non-viable) when compared to the tumor control group. These results could connote either a direct cytotoxic effect of MEAC on tumor cells or an indirect local effect, which may involve macrophage activation and vascular permeability inhibition (9).

Prolongation of the animal life span was being considered a reliable criterion for the depiction of efficacy of an anticancer agent (14). The increase of life span of tumor bearing mice by reduction of nutritional fluid volume and seization of the tumor growth is a positive result and further corroborates the antitumor effect of MEAC.

Major problems encountered in cancer chemotherapy are myelosuppression and anemia. The anemia exhibited in tumor bearing mice is mainly due to reduction of RBC or hemoglobin percentage and etiology is either iron deficiency or hemolytic/myelopathic conditions (18). Pharmacotherapy with MEAC replenishes the hemoglobin (Hb) content, RBC and WBC count to the normal levels. It is evident from the result that MEAC possess protective action on hemopoietic system.

Apoptosis was originally defined by the morphological changes that transpire with notable trustworthiness in a wide variety of cells despite of cell lineage and the method of cell death induction. Consideration of morphology is the most important method for detection of apoptosis (19). In the present experiments, apoptosis associated morphological changes were observed on DLA cells after MEAC treatment by AO/EB double staining method. It should be considered that healthy cells have green nuclei and uniformly chromatin with intact cell membrane, the cells undergoing apoptosis have orange or green nuclei with condensed chromatin and the necrotic cells have red nuclei with damaged cell membrane (16). Most of the cells treated with MEAC were apoptotic with green or orange fragmented nuclei which were also in accordance with relatively low cell viability. Our results by AO/EB double staining confirmed that MEAC could induce cell death through apoptosis.

DNA damage-induced apoptosis mainly proceeds through the mitochondrial pathway of caspase activation (20). Hence in order to understand the level of DNA damage followed by apoptosis, we had scrutinized the DNA damage by comet assay. The comet assay is a relatively fast, simple, and sensitive technique for the analysis of DNA damage in all cell types, and combines the simplicity of biochemical techniques for detecting DNA single-strand breaks, alkali-labile sites and cross-linking, with the single cell approach typical of cytogenetic assays (17). DNA damage in proliferating cells activates a pathway which arrests cell division to allow either DNA repair or induction of cell death by apoptosis. Under alkaline conditions, necrotic or apoptotic cells can result in comets with small or nonexistent head and large diffuse tails (called a ‘‘hedgehog” comet) as observed on DLA cells after treatment with MEAC (20). MEAC induced substantial DNA damage which was evident from the appearance of comet like features with considerable comet tail length (9.42 ± 1.67 and 13.17 ± 3.71 µM) at the doses 200 and 400 mg/Kg respectively.

To investigate the % of cell undergoes apoptosis of MEAC we perform double labeling techniques using Annexin-V/PI staining followed by FACS to distinguish between apoptotic and necrotic cells. Annexin V binds to phosphatidylserine (PS) in a calcium-dependent manner. PS was normally found only on the intracellular leaflet of the plasma membrane in healthy cells (19). Early apoptosis, membrane asymmetry is lost and PS translocates to the external leaflet. Fluorochrome-labeled Annexin-V then specifically target and identify apoptotic cells and excludes necrotic cells as PI unable to bind. At late stage apoptotic cells and necrotic cells will stain positively, due to the passage of these dyes into the nucleus where they bind to DNA (2). Presented flowcytometric data revealed that, increase in MEAC dose from 200 to 400 mg/Kg, severely increased in the apoptosis of DLA cells was observed suggesting that the mode of cell death is increasing apoptosis.

Apoptosis is a tightly regulated process, which involves changes in the expression of distinct genes. Bcl-2 family proteins that consist of anti-apoptotic and pro-apoptotic members are important regulators of apoptosis (1). Over expression of pro-apoptotic Bax induces the release of cytochrome c from mitochondria. Released cytochrome c contributes to apoptosis through binding Apaf-1 and caspase-9, whereas, anti-apoptotic Bcl-2 binds to Bax and can form heterodimers, which inhibit Bax activity and block apoptosis in cells. The ratio of Bcl-2 to Bax protein has been reported to be associated with apoptosis, and used as a predictive marker for therapeutic response to radiotherapy (2). Present experiment revealed that MEAC treatment to DLA cells resulted in a dose-dependent increase in the level of Bax with a concomitant decrease in Bax/Bcl-2 ration. This suggested that a critical determinant of the overall propensity of cells were undergoing apoptosis at aforementioned treatment.

## Conclusion

Present investigation is quite encouraging as it explores that methanol extract of *A. cadamba *could induce apoptosis in DLA cells through cell cycle arrest and as well as by modulation of balance between pro- and anti-apoptotic proteins. Phytochemical study confirmed the presence of compounds namely triterpenoid, glycosides, alkaloids, phenolics and flavonoids in MEAC which may possess these properties. The apoptosis inducing property of MEAC on DLA cells in animal model suggests the development of a chemopreventive agent active in cancer therapy.

## References

[1] Chatterjee S, Biswas G, Chandra S, Saha GK, Acharya K (2013). Apoptogenic effects of Tricholoma giganteum on Ehrlich’s ascites carcinoma cell. Bioprocess Biosyst. Eng..

[2] Bhattacharyya A, Choudhuri T, Pal S, Chattopadhyay S, Datta GK, Sa G, Das T (2003). Apoptogenic effects of black tea on Ehrlich’s ascites carcinoma cell. Carcinogenesis.

[3] Umachigi SP, Kumar GS, Jayaveera KN, Kishore DVK, Ashok Kumar CK, Dhanpal R (2007). Antimicrobial, wound healing and antioxidant activities of Anthocephalus cadamba. African J. Tradi. Compl. Alt. Med..

[4] Alam MA, Raushanara A, Nusrat S, Mostafizur R, Muntasir MM, Lutfun N, Satyajit DS (2008). Antidiarrhoeal property of the hydroethanolic extract of the flowering tops of Anthocephalus cadamba. Brazilian J. Pharmacog..

[5] Sianne S, Fanie RH (2002). Antimalarial activity of plant metabolite. Nat. Prod. Rep..

[6] Kapil A, Koul I, Suri OP (1995). Antihepatotoxic effects of chlorogenic acid from Anthocephalus cadamba. Phytother. Res..

[7] Kodangula SC, Borthakur A, Kodangala SP (2010). Anti-inflammatory effect of the methanol extract from Anthocephalus cadamba stem bark in animal modelsa. Int. J. Plant. Biol..

[8] Acharyya S, Dash GK, Mondal S, Dash SK (2010). Studies on Glucose Lowering Efficacy of the Anthocephalus cadamba (Roxb) Miq roots. Int. J. Pharma. Bio. Sci..

[9] Dolai N, Karmakar I, Kumar RBS, Kar B, Bala A, Haldar PK (2012a). Evaluation of antitumor activity and in vivo antioxidant status of Anthocephalus cadamba on Ehrlich ascites carcinoma treated mice. J. Ethnopharmacol..

[10] Evans WC (1996). Trease and Evan Pharmacognosy.

[11] Naskar S, Mazumder UK, Pramanik G, Bala A, Haldar PK, Islam A, Gupta M (2011). Comparative in vitro antioxidant activity of different parts of Cocos nucifera (Linn) on reactive oxygen and nitrogen species. Int. J. Pharm. Pharm. Sci..

[12] Bhattacharya S, Prasanna A, Haldar PK (2011). Evaluation of antiproliferative activity of Trichosanthes dioica root against Ehrlich ascites carcinoma cells. Academic J. Cancer Res..

[13] Dolai N, Karmakar I, Kumar RBS, Bala A, Majumder UK, Haldar PK (2012b). Antitumor potential of Castanopsis indica (Roxb ex Lindl) A DC leaf extract against Ehrlich’s ascites carcinoma cell. Indian J. Exp. Biol..

[14] Haldar PK, Kar B, Bala A, Bhattacharya S, Mazumder UK (2010). Antitumor activity of Sansevieria roxburghiana rhizome against Ehrlich ascites carcinoma in mice. Pharm. Biol..

[15] Sriram MI, Mani Kanth SB, Kalishwaralal K, Gurunathan S (2010). Antitumor activity of silver nanoparticles in Dalton’s lymphoma ascites tumor model. Int. J. Nanomed..

[16] Attari F, Sepehri H, Delphi L, Goliaei B (2009). Apoptotic and Necrotic Effects of Pectic Acid on Rat Pituitary GH3/B6 Tumor Cells. Iran. Biomed. J..

[17] Das B, Mandal S, Chaudhuri K (2014). Role of arginine, a component of aqueous garlic extract, in remediation of sodium arsenite induced toxicity in A375 cells. Toxicol. Res..

[18] Karmakar I, Dolai N, Kumar RBS, Kar B, Roy SN, Haldar PK (2013). Antitumor activity and antioxidant property of Curcuma caesia against Ehrlich’s ascites carcinoma bearing mice. Pharm. Biol..

[19] Yang S, Zhao Q, Xiang H, Liu M, Zhang Q, Xue W, Song B, Yang S (2013). Antiproliferative activity and apoptosis-inducing mechanism of constituents from Toona sinensis on human cancer cells. Cancer Cell Int..

[20] Liao W, McNutt MA, Zhu W (2009). The comet assay: A sensitive method for detecting DNA damage in individual cells. Methods.

